# Catch me if you can: free-living mice show a highly flexible dodging behaviour suggestive of intentional tactical deception

**DOI:** 10.1098/rsos.231692

**Published:** 2024-07-03

**Authors:** Raffaele d'­Isa, Michael H. Parsons, Marcin Chrzanowski, Piotr Bebas, Rafal Stryjek

**Affiliations:** ^1^ Institute of Experimental Neurology (INSPE), Division of Neuroscience (DNS), IRCCS San Raffaele Scientific Institute, Milan, Italy; ^2^ Centre for Urban Ecological Solutions LLC, Houston, TX, USA; ^3^ Faculty of Biology, Biology Teaching Laboratory, University of Warsaw, Warsaw, Poland; ^4^ Faculty of Biology, Department of Animal Physiology, Institute of Functional Biology and Ecology, University of Warsaw, Warsaw, Poland; ^5^ Institute of Psychology, Polish Academy of Sciences, Warsaw, Poland

**Keywords:** tactical deception, intentionality, *Apodemus agrarius*, cognition, cognitive ethology, naturalistic behavioral studies

## Abstract

Intentional tactical deception, the employment of a tactic to intentionally deceive another animal, is a complex behaviour based on higher-order cognition, that has rarely been documented outside of primates and corvids. New laboratory-to-field assays, however, provide the opportunity to investigate such behaviour among free-living mice. In the present study, we placed laboratory-style test chambers with a single entrance near a forest outside Warsaw, where we observed the social interactions of two territorial murids, black-striped and yellow-necked mice, under food competition for seven months. Notably, among the social interactions, we video-recorded 21 instances of deceptive pursuer evasion. In the most obvious cases, an individual inside the chamber, to avoid an incoming mouse, hid by the chamber opening (the only means to enter or exit), paused until the pursuer entered and passed by, and then exploited the distraction of the back-turned pursuer by fleeing through the opening in a direction opposite to the one the pursuer came from. This deceptive dodging is the first evidence of a behaviour suggestive of intentional tactical deception among mice. As such, this deceptive behaviour may be of interest not only for rodent psychology but also, more generally, for the fields of non-human intentionality and theory of mind.

## 1. Background

The black-striped mouse (*Apodemus agrarius*) and the yellow-necked mouse (*Apodemus flavicollis*) are two terrestrial murid rodents that share a common habitat from Central Europe to the Urals. Since the two species co-inhabit the same geographical area and are both territorial, they often engage in interspecific agonistic interactions. In particular, a specific area where the two mouse species are known to be sympatric is the forestal area outside Warsaw in Poland [1].

In the present work, we deployed free-access laboratory-style test chambers next to a forest outside Warsaw. Herein, we observed intraspecific and interspecific social interactions of *Apodemus* mice under competition for a highly palatable food, which was delivered daily in each test chamber. Between 2020 and 2023, we video-recorded a cumulative period of seven months of social interactions inside and immediately outside the chambers, and we discovered a previously undocumented defensive behaviour, performed by *A. agrarius*: a deceptive pursuer evasion, whereby one mouse inside the chamber deceives and flees from an incoming mouse. We observed two subtypes of this behaviour. In the first type, a mouse, chased by a pursuer mouse, appears to flee inside the chamber, hide laterally to the chamber opening (the only means to enter or exit the chamber), pause until the pursuer has passed, and then exploit the distraction of the backed-turned pursuer to escape in the direction opposite to the one the pursuer came from. In the second type, a mouse already in the chamber, having detected an incoming mouse, performs the same manoeuvres to exit the chamber avoiding physical contact with the intruder. Both types of pursuer evasion are consistent with intentional tactical deception, which has never been reported for mice.

Behavioural deception (i.e. performing a behaviour that sends false information or withholds true information, in a way that is beneficial to the sender and detrimental to the receiver) is present in a wide variety of animal species [2–6]. Notably, while most deceptive behaviours are instinctual and genetically predetermined, some animals employ flexible, voluntary tactics when deceiving the target. This subtype of behavioural deception, known as tactical deception, is a cognitive-behavioural manoeuvre by which the agent can flexibly transmit misinformation to another and change the other’s behaviour to its own advantage [7]. Tactical deception, requiring higher cognitive prerequisites, is rare and has been reported primarily in primates [8–10] and corvids [11–14]. Within tactical deception, the highest level is intentional tactical deception [15], in which a voluntary tactic is employed to obtain an advantage over another animal in a way that is not only deceptive but also intentionally deceptive. Among rodents, deceptive behaviours that could qualify as intentional tactical deception have, until now, only been observed in squirrels [16] and rats [17]. This is the first report of a behavioural deception suggestive of intentional tactical deception in mice.

In the current work, after describing the observed deceptive behaviour of black-striped mice, we evaluate whether this behaviour is instinctual, learned or creative, and we argue that this newly observed *Apodemus* behaviour can be considered as intentional tactical deception. This discovery could prompt us to rethink what we currently know about rodent psychology, and, more generally, it may be of interest for the fields of non-human intentionality [18] and non-human theory of mind [19].

## 2. Methods

### 2.1. Study site

The observations took place between 2020 and 2023, across two time periods: Study 1 was performed from 3 November 2020 to 9 April 2021, while Study 2 was carried out from 16 January 2023 to 25 March 2023. The observations were performed in a peri-urban area of central Poland’s Warsaw, on private lands next to a forest (52°20′20.00″N; 21°03′30.00″E; altitude of 80 m). Recording sites for Studies 1 and 2 were located 40 m apart and both sites were in close proximity to the external border of the forest. Deployment of test chambers in the private lands contiguous to the forest was authorized by the owners of the properties. Temperatures ranged from −17 to 20°C during Study 1 and from −8 to 20°C during Study 2.

### 2.2. Animals

**Figure 1.**
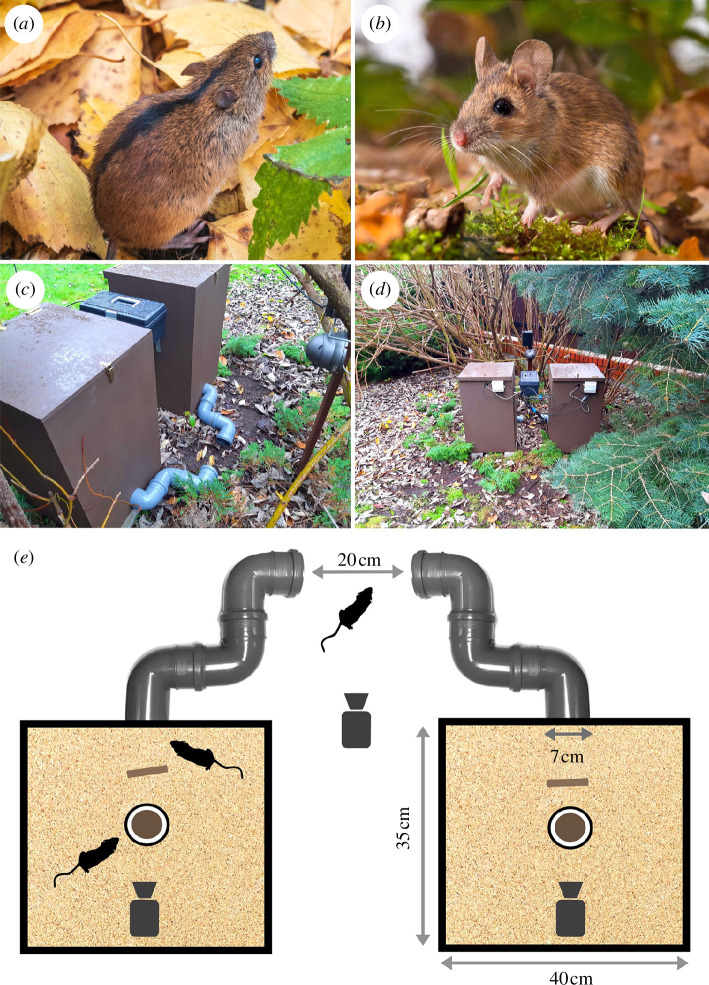
Subject species and test equipment. (*a*) Black-striped mouse (*A. agrarius*). (*b*) Yellow-necked mouse (*A. flavicollis*). (*c*,*d*) External view of the test chambers. (*e*) Schematic representation of the test chambers. Motion-activated video cameras record the behaviour of free-ranging mice outside and inside the test chambers. The brown circle at the chamber’s centre represents the bait (chocolate cream presented in a dish). The brown rectangle represents a piece of wood. The floors of the chambers are covered with sand. Photographers: Dmitry Potashkin (*a*), Rudmer Zwerver (*b*) and Rafal Stryjek (*c,d*).

The observations were carried out during a research project examining the reaction of two species of free-ranging mice, namely black-striped mice (also known as striped field mice; *A. agrarius*) and yellow-necked mice (also known as yellow-necked field mice; *A. flavicollis*) (figure 1*a,b*), to food competition in the presence of predator scents (for details see [20]). All dodges were performed in non-experimental conditions, which featured a food attractant without predator odour (21 dodges were observed during the preparatory or intertrial phases and 1 dodge occurred during a control condition). In the present work, black-striped mice are the subject animals, while yellow-necked mice represent stimulus animals.

Mice sampled in Study 1 and those from Study 2 can be considered as different populations, because more than 21 months elapsed between studies and the life expectancy of *A. agrarius* in nature is no longer than 18 months [21]. For the present study, we did not mark the animals, in order to avoid stress deriving from handling, anesthesia and surgery for microchip implantation. Only mice totally unmanipulated by humans were considered for the current study. Individual identification of dodgers was performed through visual recognition of the unique somatic characteristics of the video-recorded mice (see below).

### 2.3. Species identification

In the present study, the subject animals are *A. agrarius*, which are phenotypically identifiable beyond doubt due to the presence, on their fur, of a typical dorsal black stripe running longitudinally along their back. On the other hand, *A. flavicollis*, which bears no stripe, may sometimes be confused with another non-striped *Apodemus* species, namely the wood mouse (*Apodemus sylvaticus*), especially in the areas of southern Europe, where *A. flavicollis* and *A. sylvaticus* are phenotypically more similar [22–24].

In the recordings of our study, we did not observe any *A. sylvaticus*. However, to exclude the possibility that we could have misrecognized *A. flavicollis*, we performed genetic analysis on the tail samples of 18 non-striped mice phenotypically identified as *A. flavicollis* and collected from the area of the behavioural study (within 150 m from the recording chambers) between December 2021 and June 2023. During the 18 months of live-trapping, no mouse was phenotypically identified as *A. sylvaticus*. PCR testing revealed that, of the mice identified as *A. flavicollis* and sampled for testing, 18 of 18 were truly *A. flavicollis*, confirming the accuracy of our phenotype-based species identification (electronic supplementary material, figure S1). Importantly, these 18 mice, which were humanely live-trapped, were collected for a different molecular study. No mouse was trapped for the present study or for the sole purpose of species identification. See electronic supplementary material for a full description of the procedures of genetic testing.

### 2.4. Test chambers

Test chambers (figure 1*c,e*) consisted of wooden boxes with 35  cm × 40  cm floors and 70  cm high walls. These boxes were built with 12  mm thick waterproof plywood panels painted with odourless acrylic paint (Luxens, Leroy Merlin, France). Chambers were connected to 7  cm diameter and 50  cm long plastic sewer pipes (Certus, Cieszyn, Poland) which served as a single point of entrance or exit. Two test chambers located 20 cm apart were employed in each study. Test chambers were free-access and could be visited ad libitum by free-ranging mice at any time of the day.

An infrared video camera (Easycam EC−116-SCH, Naples, FL, USA) was placed inside each chamber, where the deceptive behaviour occurred, and one outside the chambers, where the social interaction was often initiated. To enable remote monitoring, we connected the three infrared cameras to a single digital video-recorder endowed with a motion detection system (Easycam EC−7804 T, Naples, FL, USA). This equipment allowed animal detection and subsequent recording at all hours for the whole duration of the study.

The baiting was done in the evenings, shortly after dusk, since this time period is known to reflect the peak of nocturnal rodent activity [25]. Chocolate-nut cream [26–28] was used as bait on a daily basis. In Study 1, 5  g of Nuss Milk Krem (MW FOOD, Wadowice, Poland) were delivered. In Study 2, 10 g of Nutella (Ferrero Polska, Warsaw, Poland) were delivered. The chocolate-nut cream was applied evenly on the surface of a 70  mm glass Petri dish which was placed in the middle of the chamber. The floor of each chamber was covered with 1  cm of rinsed sand and replaced after every 2–4 days. In order to eliminate possible scent markings, the entrance pipes were thoroughly cleaned with the unscented liquid soap Biały Jeleń (Pollena, Ostrzeszów, Poland) every 2–4 days.

### 2.5. Social interaction scoring

For each social encounter, the following variables were scored by visual examination of the video recordings: interspecific encounters (0 or 1), deception (0 or 1) and multistage deception (0 or 1).

### 2.6. Dodging behaviour scoring

Presence or absence of deceptive behaviour and, in cases where deception occurred, the complexity of the deceptive behaviour, were assessed by evaluating the presence or absence of the following stages of deception:

— *Hiding*. A mouse detects a cue, becomes vigilant (stands stills with its snout towards the entrance), moves away from the collision trajectory (the attack zone in red in electronic supplementary material, figure S2) and rapidly moves to a hideout zone (one of the two zones in yellow in electronic supplementary material, figure S2), with the whole body inside the zone. The tail may stick out.
— *Concealment through immobility and silence*. The first mouse (mouse 1: the chased) remains immobile, avoiding producing sounds that could reveal its position, in a hideout zone, while a second animal (the chaser) is searching for it.
— *Exploitation of the target’s distraction to escape from the tube*. Mouse 1 (the chased mouse) chooses a moment of distraction by mouse 2 (the chaser) in order to escape from the tube without physical contact. Conditions of distraction of the chaser are, for example, when the chaser: (i) has its back turned; (ii) is pointing its gaze away from the chased; (iii) is running in a direction different from mouse 1’s position, at a speed too high to be able to pivot and reach mouse 1 before it escapes; and (iv) is engaged in an action that makes it temporarily unable to react efficiently (such as grooming, eating or any other action that causes a delayed reactivity to an escape attempt of mouse 1).

This is a sequential model with step 2 omitted in the instance that, after entering, the chaser stopped too close to the hideout. This fine-tuning of the plan also requires behavioural flexibility. A behaviour was defined as deceptive dodging if at least the first deception was performed. Cases in which only stage 1 was performed were defined as basic deceptive dodges, while cases featuring more than one stage were defined as multi-stage deceptive dodges.

Deceptive behaviour was scored by five independent experienced raters. In order to evaluate the inter-rater reliability, we calculated the overall percentage of agreement among raters (i.e. the overall mean of the percentage of raters in agreement for each dodge) and Fleiss’ kappa for inter-rater reliability [29]. The overall percentage of agreement among raters was 98.8%. Fleiss’ kappa for inter-rater reliability was 0.96. In all cases, no less than 4 of 5 raters agreed on the score.

### 2.7. Statistical analysis

The association between the variable type of social encounter (intraspecific or interspecific) and the variables deception (present or absent), multistage deception (present or absent) and fight (present or absent) was analyzed through Fisher’s exact test. The association between the variable population (1 or 2) and the variables type of social encounter, deception, multistage deception and fight was analyzed through the same method. Significance level was set at *p* < 0.05. Statistical analysis was performed with the software IBM SPSS Statistics 23.

### 2.8. Individual identification of dodgers

Based on the size, the coat pattern and the population of origin of the mice, we were able to positively identify five different individuals among the dodgers. Since only a minority of individuals bear somatic characteristics allowing distinctive identification, we estimate that the total number of dodgers is much higher, probably about 12–15 individuals.

### 2.9. Computer graphics reconstruction of dodging behaviour dynamics

**Figure 2.**
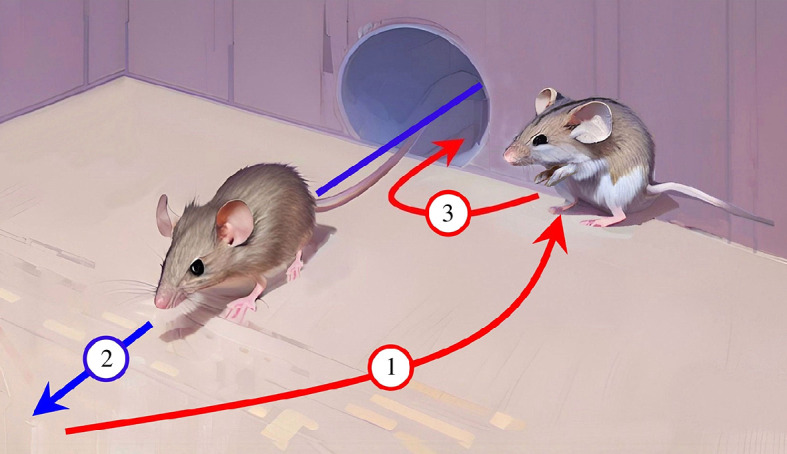
Graphical representation of the essential stages common to both types of deceptive dodge. Actions performed by the chased mouse and the chasing mouse are represented in red and blue, respectively. (1) Hiding: one mouse is inside the chamber, in front of the tube, and, upon hearing an incoming second mouse, it moves away from the interception trajectory, reaching a safe spot lateral to the tube. (2) Concealment through immobility and silence: after the chaser enters the chamber and is deceived by finding the chamber seemingly empty, the chased mouse remains immobile, avoiding producing sounds that could reveal its position, in a hideout zone, while the chaser is looking for it. (3) Exploitation of the target’s distraction: the chased mouse exploits the chaser mouse’s distraction and the entrance tube’s availability to escape, avoiding a fight.

Computer graphics representation of the dynamics of the dodging behaviour (figure 2) was created with Microsoft PhotoDraw 2000 v.2 and Wonder—AI Art Generator. More than 30 photos of black-striped mice and of the chamber were used as input to instruct the artificial intelligence software.

## 3. Results

### 3.1. Description of a new defensive behaviour observed in *Apodemus* mice: deceptive dodging

Social interactions of wild free-ranging *Apodemus* mice (figure 1*a,b*) were observed between 2020 and 2023 through video camera-equipped test chambers (figure 1*c*–*e*) placed near a forest in a peri-urban area of Warsaw in Poland. We performed observations across two periods. Study 1 was carried out from November 2020 to April 2021. Subsequently, in order to confirm our results in a different population of mice, we collected data through Study 2 from January 2023 to April 2023. Importantly, since the life expectancy of *A. agrarius* in nature is no longer than 18 months [21] and since the inter-study period was more than 21 months, we can consider the mice sampled in Study 1 and the ones in Study 2 as two different populations of individuals.

**Figure 3.**
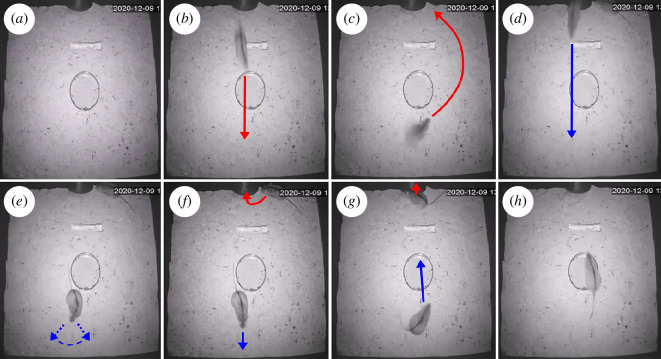
Dynamics of a type A deceptive dodge. (*a*–*h*) Sequential snapshots depicting the dynamics of a type of deceptive dodge performed by a black-striped mouse. Actions performed by the chased mouse and the chasing mouse are represented in red and blue, respectively. The integral video is shown in electronic supplementary material, video S1.

Recordings revealed a very peculiar behaviour performed by black-striped mice as defense against heterospecific and conspecific individuals. In a prototypical situation, a mouse (the chased) is pursued by another mouse (the chaser) outside the chamber and enters the chamber to evade its pursuer, where it performs a manoeuvre which we define as *deceptive dodge*. Figure 3 shows, through sequential snapshots, the dynamics of this deceptive dodge. Initially, the chamber is empty (figure 3*a*). A mouse which is being chased outside the chamber enters the chamber at high speed (figure 3*b*). Once it has reached the centre of the wall facing the tube (the southern wall), the chased mouse, hearing that the chaser mouse is coming from the tube, turns and rapidly moves away from the collision trajectory of the incoming mouse, moving towards a specific spot lateral to the tube (figure 3*c*). The chaser mouse enters the chamber and crosses the central spot (figure 3*d*). The chaser mouse halts in front of the southern wall, as if confused by a mismatch between the expectancy of finding the chased mouse and the perception of an empty chamber (figure 3*e*). The chaser mouse looks left and right, apparently seeking the chased mouse, while the chased mouse remains immobile and avoids producing sounds and floor vibrations that could reveal its position (figure 3*e*). As soon as the chaser mouse resumes forward movement, the chased mouse immediately flees towards the exit (figure 3*f*). The chaser mouse turns and apparently detects that the chased mouse is at the exit and about to leave the chamber, after which it tries to reach the chased mouse (figure 3*g*). The chaser mouse crosses the chamber, but the advantage cumulated by the chased mouse is too substantial, so when the chaser mouse arrives at the centre of the chamber, the chased mouse has already fled (figure 3*h*).

The necessity of such an elaborate and deceptive scheme for escape is derived from the fact that the tube connected to the chamber is the only route of entry or exit. It is not possible to exit the chamber if there is another mouse in the tube. In order to avoid a fight, the only possible way to escape is to harness the moments, after the entrance of the chaser mouse, when the tube is free and the chaser mouse has its back towards the entrance/exit, to take the tube and leave the chamber.

**Figure 4.**
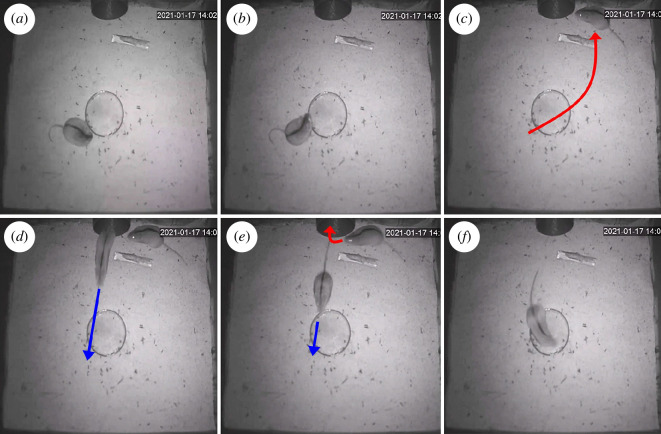
Dynamics of a type B deceptive dodge. (*a*–*f*) Sequential snapshots depicting the dynamics of a type B deceptive dodge performed by a black-striped mouse. Actions performed by the chased mouse and the chasing mouse are represented in red and blue, respectively. The integral video is shown in electronic supplementary material, video S2.

In a second prototypical situation, the chased mouse is already in the chamber, alone (figure 4*a*). Upon detecting the sound of an approaching mouse in the entrance tube, the first mouse displays a sudden orienteering response, directing the head towards the source of the sound, the tube (figure 4*b*). The first mouse runs to a safe spot, reaching it before the second mouse enters (figure 4*c*). The second mouse, the pursuer, enters the chamber and finds no mouse inside (figure 4*d*). The first mouse exploits the disadvantageous position of the second mouse (back towards the tube and snout towards the southern wall) to exit the chamber (figure 4*e*). When the second mouse has reached the chamber’s centre, the first mouse has already fled (figure 4*f*).

Figure 2 summarizes the essential stages common to both prototypical situations, which we define, respectively, type A and type B. Both dodge types A and B comprise three stages: (i) hiding; (ii) concealment through immobility and silence; and (iii) exploitation of target’s distraction. Description of the stages is provided in figure 2.

### 3.2. Analysis of frequency and complexity of deceptive dodging

Deceptive dodges were a rare event. Overall, we recorded 143 social encounters (interspecific or intraspecific) featuring black-striped mice. From this total number of social encounters, we observed black-striped mice performing deceptive dodging behaviour in 21 cases (14.7%). Additionally, we also report deceptive dodging in the yellow-necked mouse, but this appeared to be an exceptional case. We recorded 64 social encounters (interspecific or intraspecific) featuring yellow-necked mice. Among this total, we observed yellow-necked mice performing deceptive dodging behaviour in only 1 case (<0.02%). For this reason, we focused on the black-striped mice as the subject of the study. Of the 143 social encounters of black-striped mice, 121 (84.6%) were intraspecific. On the other hand, deceptive dodges of black-striped mice were intraspecific in only 13 of 21 cases (61.9%). Interestingly, while in intraspecific encounters, black-striped mice performed deceptive dodging in only 10.7% of the cases, in interspecific encounters they executed deceptive dodges with a frequency more than three times higher, 36.4%, suggesting that the deception employed by black-striped mice is mainly an interspecific defensive strategy. The main target of interspecific deception (87.5% of the cases) were yellow-necked mice, which are bigger, stronger and dominant over black-striped mice. Notably, contingency table analysis through Fisher’s exact test revealed a significant association between the variable deception (0 = absent; 1 = present) and the variable interspecific encounter (0 = no; 1 = yes), with deception occurring higher than expected in interspecific encounters (*p* = 0.005).

Concerning the dodge type, 19.0% of the dodges were type A (subject mouse entering the chamber to escape from a pursuer after a chase initiated outside the chamber) and 81% were type B (subject mouse inside the chamber when a second mouse entered the tube).

The level of complexity of the deception varied across the deceptive dodges. Over the total number of deceptive dodges, the majority (12; 57.1%) were simple dodges in which only the first stage of the deception described in figure 2 was performed. Subsequently, the deception of the first mouse was interrupted for one of the following reasons: (i) the mouse in the tube, instead of entering the chamber, moved back outside the tube; (ii) the mouse in the tube entered the chamber, but turned out to be non-hostile, so the first mouse pacifically interacted with it; and (iii) the mouse in the tube entered the chamber and spotted the first mouse in the hideout zone, attacking it. Notably, on nine different occasions (42.9%) we observed complex multistage deceptions carried out by the first mouse. Overall, stage 1 was performed in all cases, stage 2 in 4 cases (19.0%) and stage 3 in 8 cases (38.1%). While in intraspecific encounters complex multistage deceptions were performed just in 3.3% of the cases, in interspecific encounters they were performed in 22.7%. Fisher’s exact test revealed a significant association between the variable multistage deception (0 = absent; 1 = present) and the variable interspecific encounter, with multistage deception occurring higher than expected in interspecific encounters (*p* = 0.005).

Between the two populations of black-striped mice studied, no difference was observed in the frequency of deception or of multistage deception. Fisher’s exact test found no association between the variable population and the variable deception (*p* = 0.631), nor between population and multistage deception (*p* = 0.312). Frequency of interspecific encounters also appeared to be comparable in the two populations (Fisher’s exact test, *p* = 0.814).

Complete results are included in electronic supplementary material. Population sampled, date of event, species involved, dodge type and complexity of deception for each case are reported in electronic supplementary material, table S1. Deception stages for each deception case are reported in electronic supplementary material, table S2.

## 4. Discussion

### 4.1. Defensive dodging of black-striped mice as a form of deception

This newly documented defensive behaviour of black-striped mice can be classified as pursuer evasion, since the evading mouse actively escapes an approaching mouse in order to avoid physical contact and a possible fight. More specifically, the pursuer evasion strategy adopted by black-striped mice appears to conform to behavioural crypsis (a behaviour reducing the likelihood of detection), which is a form of behavioural deception [2–5]. Deceptions can feature simulation (showing the false) or dissimulation (hiding the true) [30]. In our case, the observed deception belongs to the second category. Indeed, the behavioural crypsis of black-striped mice is deceptive in the sense that, by withholding information, it dissimulates the position of the performer, to the disadvantage of the target and to the benefit of the performer.

As a first means of defense, an active hiding behaviour is employed by black-striped mice to prevent detection by the pursuer. Hiding behaviour is the action of changing position in order to reduce the likelihood of being detected. In our observations, black-striped mice ostensibly exploit the darkness of the chamber to reduce the likelihood of being seen (visual crypsis), and within the chamber, they choose a tactical position to hide, one of the two zones lateral to the tube, a location that, after entrance of the pursuer, will allow them to be behind the pursuer, in a position outside the pursuer’s visual field, which makes the chamber appear empty to the target of the deception.

Most interestingly, in many cases, the deception of black-striped mice is not limited to simple hiding, but is actually a complex multistage behaviour which continues with two further stages of deception. After the second mouse has entered the chamber, the first mouse remains immobile, avoiding producing sounds that might reveal its position (acoustic crypsis) and avoiding generating floor vibrations (tactile crypsis). We defined this second stage as concealment.

Finally, in the third phase of the deception, the first mouse (the chased) monitors the behaviour of the second mouse (the chaser) and, when the occasion allows it, it exploits the distraction of the second mouse to escape through the tube. This complex multistage scheme of deception, composed of hiding, concealment and distraction exploitation, prompts questions on its origin.

### 4.2. Is the behavioural crypsis of black-striped mice instinctual, learned or creative?

On the basis of their origin, pursuer-evasion behaviours can be subdivided into three main types: instinctual, learned or creative. Let us describe the characteristics of each of these three types of evasion behaviour and evaluate which one fits best the behaviour of the black-striped mice observed in the test chambers.

Instinctual behaviours are innate, genetically determined behaviours that promote the survival of the individual and/or of its species. An example of instinctual evasion behaviour is anti-predatory freezing [31,32], which in particular is a form of behavioural crypsis. Many rodents, in the presence of terrestrial predators (felids or canids), exhibit freezing behaviour, i.e. cessation of all voluntary movements. Freezing is an adaptive behaviour, as for predators it is more difficult to distinguish from the background a still target rather than a moving target. In laboratory, a common paradigm to study anti-predatory freezing is the predator odour test [33–36]. Interestingly, if laboratory mice are exposed to the sole odour of a predator, they commonly display freezing behaviour. Importantly, this freezing reaction is exhibited even in the absence of an actual predator, even if the mouse never experienced an encounter with that predator and even if the mouse has been bred from mice that never encountered a predator. These facts suggest that anti-predatory freezing is instinctual. The absence of an actual predator representing an evident threat rules out the hypothesis of a creative goal-directed behaviour. On the other hand, the lack of previous predator encounters rules out learning and acquired conditioned responses. Furthermore, anti-predatory freezing is displayed by the vast majority of mice presented with predator odour and it is manifested in an extremely stereotyped form, totally similar across individuals of the same species. These two features further exclude creative behaviour, which by definition is unique, stochastic and variable across individuals.

Regarding the behavioural crypsis that we observed in black-striped mice, the simplest explanation would be that the behaviour is instinctual. However, several facts make this hypothesis unlikely. First, mice were tested in an apparatus (the test chamber) with a design created by a human and which is not a specific object of the natural world of the mice, an object in response to which evolution could not have provided specific instincts. Mice began their evasive strategy when no other mouse was present in the chamber, ostensibly at the sound of another mouse arriving from the tube. This highly specific sound of another mouse running in the tube is never heard in a natural environment (the tubes are man-made objects) and cannot have been selected during the natural evolution of black-striped mice as an auditory stimulus triggering an instinctual defensive behaviour (contrarily to freezing in response to predator odour). Second, instinctual behaviours are largely shared across individuals of the same species, while creative behaviours are rare. In our recordings, on 143 total social encounters, we observed black-striped mice performing deceptive dodging in just 21 cases (14.7%), indicating that indeed this is a rare behaviour. Third, instinctual responses are stereotyped, not flexible on the basis of the situation and extremely similar between individuals of the same species. In our observations, the temporal sequence of the evasive actions (from leaving the central zone of the chamber to escaping through the tube) was not fixed, but rather regulated on the basis of the situation. Indeed, after reaching the area lateral to the tube, the first mouse did not immediately take the tube to escape, even though the exit was just nearby. Rather, the first mouse stopped next to the tube, as if planning to use it at a more appropriate moment. The hiding mouse monitored the behaviour of the chaser and waited for the most appropriate moment to exit, deciding to escape through the tube only when the chaser mouse entered the chamber and, being turned or otherwise distracted, could not react efficiently. If taking the tube was an instinctual response to a stimulus associated with an intruder (for instance, an instinctual escape response similar to entering a hole), then it should have been performed immediately, when the stimulus was detected, not with such a long and variable delay. In contrast, in the observed cases, the mouse in the chamber moved close to the tube, but without entering it, and used the tube only when the situation was most appropriate for an escape. This fine-tuning of the evasive behaviour exhibited by the first mouse suggests that the behaviour is not a fixed, stereotyped, instinctual response, but rather a behaviour voluntarily regulated to achieve a specific goal (avoiding getting reached).

Apart from the instinctual hole-entering response, other instinctual defensive responses that could have been employed by a mouse in the chamber upon detection of an incoming mouse are the innate flight response and thigmotaxis. However, both of these possibilities appear to be incompatible with the observed evasive behaviour. Regarding the possibility of an instinctual flight, mice are endowed with innate flight responses to specific stimuli. For instance, a rapidly looming dark disc over the head of the mouse (which resembles the approaching figure of an aerial predator, such as a hawk or an owl) readily triggers flight in mice, even if they are laboratory mice which never experienced an encounter with an aerial predator in their entire lives [31,32,37,38]. However, this basic innate flight does not correspond to the characteristics of the evasive behaviour we observed. The innate flight behaviour is a basic, instinct-mediated, escape response consisting of getting far from a cue which signals danger. In particular, in sound-induced innate flight responses, mice consistently move to the side of the testing chamber which is farthest from the sound [39]. If the evasive behaviour we observed was such an innate flight response, then the first mouse in the chamber would have moved far from the tube entrance, likely to the southern wall (the wall facing the tube) or in one of the two corners of the southern wall, which are the two points most distant from the tube entrance. In contrast, mouse 1 moved in the opposite direction, i.e. towards the tube, as if planning to use the tube in a future moment when the situation would have made it safer to take the tube (taking the tube while a hostile mouse is still inside it would lead to a fight). Finally, thigmotaxis (also known as wall hugging) is the tendency of rodents to avoid open spaces and stay close to vertical surfaces, especially in situations of anxiety [40–42]. The hypothesis of an instinctual thigmotactic reaction to the danger cue can be ruled out as well. Indeed, in the case of a thigmotactic response, mouse 1 would have moved randomly towards any wall. In contrast, the dodging mice moved specifically to one of the two zones lateral to the tube, sometimes even alternating them while waiting for the arrival of the intruder. Moreover, during thigmotactic behaviour, mice keep at least one flank in contact with or very close to a wall. On the contrary, in our recordings we observed that, after the dodging mouse moved to one of the two zones lateral to the tube, often its flank was not kept in contact with the wall or not even close to it (often the body of the mouse was not even parallel to the wall, but rather perpendicular to the northern wall).

If the deceptive behaviour of black-striped mice is not instinctual, an alternative hypothesis could be that it is learned. In this case, specific locations within the chamber could have been associated, in previous social encounters, with fights and could have hence generated conditioned place avoidance. In particular, through operant conditioning [43], in which behaviours are shaped by punishments and rewards, the act of moving to unsafe zones would be punished by a physical aggression leading to a fight, which would reduce the likelihood to repeat the same choice. Hypothetically, moving to the hideout zone (which is safer) could result just from the exclusion of the unsafe places, after a trial-and-error process. Interestingly, the deceptive evasion we recorded does not appear to be learned either. First, deceptive dodges were observed starting already from the first period after the test chambers were placed in the natural environment of the mice, in a phase when the mice had little or no experience of the chambers. Second, fight events in the chambers occurred, but their number was too small to support conditioning learning. Third, the evasive behaviours appeared before the fights. In Study 1, deceptive dodging was first observed on day 7, when 0 previous fights had occurred. Analogously, in Study 2 the first deceptive dodge was observed on day 10, when 0 previous fights had occurred. This excludes the possibility of place conditioning. Fourth, if the deceptive evasions were conditioned behaviour resulting not from multiple punishments (exclusion process) but from one single negative reinforcement (cessation of a fight upon reaching a certain spot), then the target place for hiding should be a specific place reinforced by a specific previous situation. On the contrary, mice alternated randomly the choice of the hiding position, even within the same situation. Hiding laterally to the tube allows two positions: the left and the right. Upon detection of an incoming mouse in the tube and after hiding, if enough time elapsed without the entry of an intruder, some mice moved from one lateral hideout zone to the other. For instance, as can be seen in electronic supplementary material, video S2 (Example 2), a mouse, after choosing one hideout zone, amazingly alternated side three times, demonstrating constant vigilance and an extremely flexible behaviour.

An alternative possibility is that the mice could have learned the dodge manoeuvre from previous experiences not inside but outside the test chamber. This would require previous experience in a structure with spatial characteristics similar to those of the test chamber. In nature, such structures with a chamber connected to a single tunnel for entrance and exit are uncommon, but they can occasionally be found in the underground living environment of rodents. Hence, a hypothesis could be that the mice in the test chambers perform the dodge as an automatic escape response learned through operant conditioning in their underground burrows, which feature tunnels. Indeed, black-striped mice, unlike some other terrestrial rodents, are burrowing animals [44–46], meaning that they dig and inhabit underground burrows where they form a nest. The underground nesting chambers are connected to the surface by tunnels. It can be hypothesized that the dodging mice could have learned their dodging manoeuvre when they were in such burrows and stranger animals invaded them. In support of such a hypothesis, a study that employed an automated event-recording system, based on passive integrated transponders carried by the subjects, showed that mice may often visit the burrows of others [47]. In particular, in this study, 1452 mice were tagged with transponders and their behaviour in nature was monitored. Interestingly, the results showed that, while most burrows (>250) were visited by only one mouse, almost 200 burrows were visited by 2 mice, a little more than 100 by 3 mice, slightly more than 100 by 4, about 80 by 5, about 70 by 6, about 60 by 7 and about 50 by 8. Considering the results not in terms of mice per burrow, but of burrows per mouse, it was found that, while the relative majority of mice visited only one burrow (21%), the remaining 79% visited multiple burrows (2: 18%; 3: 16%; 4: 16%; 5: 9%; 6: 8%; 7: 3%; 8: 3%; <8: 6%). However, it is important to note that the burrows of *Apodemus* mice are generally multi-tunnel burrows that do not feature the single entrance/exit tunnel situation [48]. Examination of the burrow structure of *Apodemus* mice revealed that the vast majority of burrows had between 2 and 5 different entrances and that, over five different study sites, for each site, the average number of entrances of a burrow was between 2 and 4. This makes it much less likely, although not impossible, that the dodging mice could have previously learned the dodge in a single-tunnel burrow. It is already unlikely that the mice had had previous experiences in single-tunnel burrows, but the learning-in-burrow hypothesis would additionally require a series of sequential conditions. In particular, the following events should have occurred: (i) a mouse should have lived in a single-tunnel burrow (which could have been a finished single-tunnel burrow or a complex burrow still under construction, in its early stage when it temporarily featured only the first tunnel); (ii) the resident mouse should have been visited by a stranger exactly in that period; (iii) the stranger should have been hostile; (iv) the resident should have performed a dodge in the nest chamber; (v) the dodge should have been successful in avoiding a fight with the intruder; (vi) an efficient reinforcement of the dodging behaviour through a negative reward (fight avoidance) should have been established; and (vii) the mouse should have kept memory of this association until the event in our testing chamber.

Most importantly, even if there had been previous learning in a single-tunnel burrow, it is unlikely that an automatic stimulus–response (S-R) conditioning could explain the dodging behaviour we observed in the chamber. If the dodging behaviours in our test chamber were a simple S-R conditioned reaction, then the stimulus (S) would be the sound of an incoming animal and the motor response (R) would be moving aside of the tunnel entrance. However, the auditory stimulus triggering the conditioned behaviour in the test chamber would be very different from the stimulus that formed the S-R association in the burrow. One of the fundamental principles of conditioning is the generalization gradient, according to which, in an S-R bond, the likelihood of the performance of the response progressively decreases with an increase in the distance between the sensorial characteristics of the trained stimulus S1 and a new stimulus S2 [49–51]. In the current case, the difference between S1 and S2 would be very wide, as the sounds of a mouse running in a plastic tube or in a ground tunnel are completely different, under every aspect of auditory perception (pitch, timbre and loudness), to an extent that it is unlikely that the original CS stimulus could be generalized through simple implicit conditioning. Rather, a successful generalization would be based on generalization of the whole situation, which would require first an explicit evaluation of the contingent situation, subsequently a recall of similar past situations and finally an action planning based on past information but flexibly adapted to the contingent context. Notably, if the mouse employed previous information to creatively develop, based on analogy with a similar past situation, a new solution in a new context for a specific goal (which is a much higher form of cognition than implicit S-R conditioning), then this would represent a form of intentional tactical behaviour.

In conclusion, both in the case that the dodging mouse expressed an entirely creative behaviour or in the case that the mouse used in part previous information acquired through experiences in the burrows to develop a solution for a contingent problem, the behaviour of the mouse would be goal-directed and intentionally tactic. Therefore, the likeliest hypothesis is that the deceptive dodges expressed by the mice may draw in part on an instinctual behaviour present in the natural ethogram of the species (thigmotaxis induced by a signal of threat) and, at the same time, may be to some extent modulated by learning (namely operant conditioning taking place in single-tunnel burrows). However, the deceptive dodges seem to go beyond instinctual or conditioned predetermined behaviour and employ past information flexibly to creatively develop a new solution that is adapted to the contingent situation with goal-directedness. Hence, the stepping aside of the tube when chased would not be driven by instinct or by operant conditioning, but would rather be a voluntary action with a specific goal.

To sum up, deceptive dodging appears to rely not merely on instinct or on learning, but rather on creative behaviour. Importantly, by creative behaviour, we are not implying that the dodging mice were not using at all any piece of information acquired in the past, but rather that the mice were combining pieces of past information acquired in other circumstances into a new solution appropriate for the achievement of a specific goal in the current circumstance. Indeed, it is unlikely that the entire motor sequence of the deceptive dodging could have been a preprogrammed behaviour acquired through operant conditioning. Rather, the dodging appears to be a highly flexible behaviour based on online decision making and tailored to the situation of each specific case in a way that optimizes the chances to achieve the goal. Previous conditioning in the chamber is incompatible with the recorded evidence. Indeed, the complex spatial characteristics of the testing chamber in relation to the tube and the very specific sound of a mouse running in the tube are stimuli that would have required experience for a specific conditioning. However, both the structure of the chambers and the situation of an agonistic encounter inside the chamber were still new when the deceptive dodges were observed and the appearance of deceptive dodging was preceded by zero fights. On the other hand, previous conditioning outside the chamber (specifically, in the subterranean burrows) cannot be excluded, but seems unlikely due to the degree of difference between the triggering stimuli and the general contexts of the burrow and the test chamber situations, a level of difference which appears too big for the maintenance of an automatic S-R response. Finally, the temporal sequence of the evasive actions, as well as the spatial targets, were different across mice and across situations, suggesting creative behaviour based on online decisions and not behaviour predetermined by instinct (which should be the same across individuals of the same species) or by conditioning learning in an underground single-tunnel burrow (which would be an environment shared across the individuals and should hence generate a more coherent conditioned behaviour).

### 4.3. Is the dodging behaviour of black-striped mice simple behavioural deception or intentional tactical deception?

High-level deception in non-humans was formally defined in the 1980s mainly by three behavioural scientists: Robert Mitchell, Richard William Byrne and Andrew Whiten.

Mitchell subdivided deception into four levels [52]. First-level deception is genetically predetermined, involuntary and displayed in every condition (examples are structural or chromatic mimicry). An example is provided by beetle daisies, which evolved floral spots mimicking the appearance of female bee flies and, through sexual deception, attract mate-seeking male bee flies from which they obtain pollination [53–55]. Second-level deception is also predetermined and involuntary, but it is performed in response to a specific stimulus. An example is the instinctual anti-predatory freezing of mice in response to predator odour [33–36]. Third-level deception includes deceptive behaviours learned through operant conditioning. Although these behaviours are intentional, they are not intentionally deceptive. Hence, no intentional deception is present in these cases. Finally, fourth-level deception refers to voluntary behaviours performed with the intention to deceive a target.

On the other hand, Byrne and Whiten introduced the concept of tactical deception [8,56], which they defined as ‘acts from the normal repertoire of the agent, deployed such that another individual is likely to misinterpret what the acts signify, to the advantage of the agent’ [56]. They described tactical deception as a flexible and voluntary behaviour [57]. Tactical deception can be distinguished from non-tactical deception based on the fact that it requires a certain degree of cognitive elaboration. As reported by Byrne, non-tactical deception corresponds to Mitchell’s first and second levels of deception while tactical deception corresponds to Mitchell’s third and fourth levels of deception [15]. More specifically, tactical deception can be further divided into non-intentional and intentional. Non-intentional tactical deception, which requires cognitive elaboration and is voluntary but not intentionally deceptive, corresponds to Mitchell’s third level [15]. An example is deception learned through a process of operant conditioning, such as, for example, the so-called rain dance of seagulls, a behaviour also known as foot-paddling [58–62]. Moles commonly consume earthworms, which represent a major component of their diet. When a digging mole is approaching, worms detect the vibrations produced by the digging activity and start emerging to the surface to escape the predator [62]. Seagulls, which occasionally may also prey on worms, employ a deceptive strategy to catch the worms. In particular, seagulls repeatedly tap the ground with one or both feet to make the worms come to the surface, where they can eat them [58–62]. By foot-paddling, gulls produce vibrations that mimic the arrival of an underground predator and prey on the emerging worms. This type of behaviour is voluntary and deceptive but not intentionally deceptive, as seagulls have simply associated the motor pattern of earth-tapping with the appearance of a food reward. On the other side, intentional tactical deception, which is the highest form of deception and corresponds to Mitchell’s fourth level, is intentionally deceptive, which requires a theory of mind and second-order intentionality [15].

**Figure 5.**
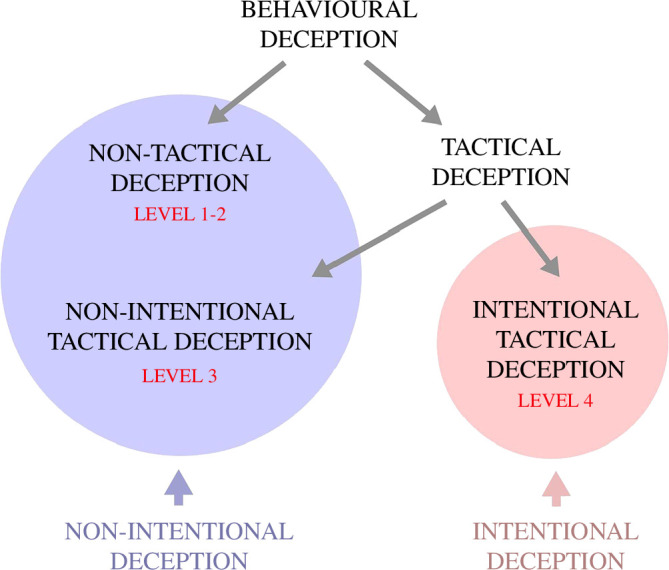
Categorization of behavioural deception. The graph shows the subdivisions of behavioural deception, with the corresponding Mitchell’s levels of deception.

Behavioural deception, which is a general category including any deception induced by a behaviour, can hence be subdivided into non-tactical (or simple) and tactical. Additionally, behavioural deception can also be subdivided into non-intentional behavioural deception (which includes non-tactical deception and non-intentional tactical deception) and intentional behavioural deception. Figure 5 summarizes the subdivisions of behavioural deception. While non-intentional behavioural deception is determined by instinct and conditioning, intentional tactical deception is instead based on goal-directed decision making aimed at deceiving a target. Compared to simple behavioural deception, tactical deception, requiring higher cognitive prerequisites, is much rarer in the animal kingdom. Intentional tactical deception is even rarer. For example, among rodents, behaviours possibly qualifying as intentional tactical deception have, at present, been reported only in squirrels, in the context of food caching simulation [16] and dissimulation [63–65], and rats, in the context of hide-and-seek play [17,66,67].

What kind of deception does the deceptive dodging of black-striped mice belong to? We submit here that the deceptive dodging of black-striped mice is highly suggestive of intentional tactical deception or Mitchell’s fourth level of deception. We examined in §4.2 several reasons for which we exclude the explanation of black-striped mice’s deceptive behaviour through instinct (which would be Mitchell’s second level) and through conditioning learning (which would be Mitchell’s third level). Exclusion of instinct and learning strongly suggests that deceptive dodging is creative. Creative behaviour can be random or goal-directed. Our observations showed strong evidence of goal-directed behaviour. In both dodge type A and dodge type B, if the first mouse inside the chamber was simply motivated by instinctual fear, upon detection of an intruder coming from the tube, it should have moved far from where it detected the intruder, that is, far from the entrance of the chamber, likely next to the southern wall or in one of the two corners of the southern wall, which are the most distant points from the entrance of the chamber. In contrast, the chased mice showed the opposite behaviour: they moved towards the tube. There, the mice stopped next to the tube, as if planning to use it at an appropriate moment, which they actually did, after the chaser entered the chamber and got far from the tube. It appears that moving next to the tube is part of a voluntary tactic employed by black-striped mice to deceive the incoming mouse by withholding information regarding their position, and to use the tube to escape when the incoming mouse is unable to react efficiently.

In particular, both the place for hiding and the duration of hiding were not without purpose, but were rather chosen tactically. The chased mice never chose a position in front of the chaser mouse to hide, but rather they chose positions that would have kept them behind the back of the incoming mouse after its entrance. Importantly, in addition to the relative position of the chased with respect to the chaser, also the specific place chosen as hideout zone was not random. Indeed, the chased mice moved away from the collision trajectory of the incoming mouse and hid specifically in one of the two areas lateral to the entrance/exit tube, which, on the one hand, are places that are out of the way of the possible roaming about of the chaser when it would have been in the chamber, and, on the other hand, are also blind spots, i.e. places from which the chased mice are more difficult to detect. Notably, mouse 1 did not choose the place to move based on the current position of mouse 2, but on the forthcoming position of mouse 2. This indicates an ability to predict a future scenario and is suggestive of a high goal-directedness in the behaviour of mouse 1. Moreover, the timing of the actions of the chased mice was not fixed, but rather showed a fine-tuning and a choice of the most appropriate moment when to act. Indeed, instead of approaching the exit as soon as the way was free for escape, the chased mice remained immobile avoiding to produce sounds and vibrations that could reveal their position and they monitored with attention the behaviour of the chaser mouse. Only when the chaser mouse had entered the chamber and was turned with the head in front of the southern wall (the wall opposite to the tube), the chased mouse took the tube to exit the chamber, indicating that the duration of the permanence in the hiding location was not random, but rather decided on the basis of the behaviour of the chaser, revealing a monitoring of the target’s position and a clear intent to avoid physical contact with the chaser. Interestingly, mouse 1, in order to choose a safe spot to hide, should understand where mouse 2 would most likely search. This, together with the fact that mouse 1 systematically exploits the disadvantageous position of mouse 2 (being turned) to take the tube, suggests an at least elementary form of perspective taking. As a whole, the behaviour of the chased mouse appears to be functional in deceiving the target, as it makes a chamber appear empty when it is not, which represents an instance of misinformation through withholding of true information [30,68–70]. Such a goal-directed creative behaviour aimed at misinforming a target is exactly what qualifies as intentional tactical deception (Mitchell’s fourth level).

Notably, performing deceptive dodging as an intentional tactical deception implies several underlying cognitive abilities, in particular: (i) sound detection (to hear the sound from the tube); (ii) sound recognition (to recognize this sound as produced by the running of another mouse); (iii) mental imagery (to imagine an approaching mouse); (iv) motion trajectory prediction (to predict in which position the incoming mouse will arrive); (v) forethought (to imagine the consequences of a possible encounter); (vi) problem-solving, decision making and planning (to choose the hiding strategy); and (vii) theory of mind (to imagine from which position it would be hardest to be detected by the incoming mouse). Altogether, these cognitive requisites reveal high-order cognition and complex social decision making in black-striped mice.

### 4.4. Usefulness of naturalistic ethological paradigms in future behavioural neuroscience research

The dodging behaviour we observed in *Apodemus* mice is spontaneous (not instructed by the experimenter) and performed by animals that were born and lived freely in nature (not captive). Test chambers were placed in the natural habitat of *Apodemus* mice and each animal could choose if, when, and for how long to explore the chamber, which makes this behavioural test strongly based on an ethological approach. Indeed, experiments adhering to what has recently been defined as ethological neuroscience not only maximize the animal welfare of experimental subjects and increase the reproducibility of experimental results by reducing stress-induced variability [71], but may also lead to the discovery of behaviours that would not be possible to observe in standard laboratory settings for behavioural testing. An example is a peculiar behaviour, named tail-belting, performed by *Apodemus* mice in sub-zero conditions to prevent tail frostbite, which has been discovered for the first time only recently through the employment of video-monitored free-access test chambers [72].

By using such a naturalistic behavioural paradigm, we found a type of behavioural deception that represents the first case suggesting intentional tactical deception in mice and that we consider one of the strongest pieces of evidence up to now of intentional tactical deception in rodents. Rodent intentional tactical deception has been, up to the present, proposed only for squirrels and rats. Notably, in relation to previous studies on rodent deceptive behaviours, our report of intentional tactical deception in black-striped mice is the first rodent case that has been observed to be performed: (i) spontaneously (not instructed by humans); (ii) in nature; (iii) by free-living animals; and (iv) directed to a specific target individual that we document being effectively deceived by the emitter of the deceptive behaviour during a direct and dynamic social interaction (a manoeuvre of evasion from a chaser).

However, it is important to underline that we consider intentional tactical deception in mice as a scientific hypothesis, not as a fact. Trying to understand intentions in mice, and in non-human animals in general, is a particularly hard quest. Indeed, non-human animals do not speak and we make our inferences regarding their psychological states mainly relying on observable overt behaviours. Nevertheless, as stressed by philosophers of mind, science has an epistemological limit to this. In fact, due to the so-called epistemological problem of other minds [73–77], it is impossible to demonstrate intentionality not only in mice but also in any other non-human animal species, and, in theory, also in humans different from ourselves. For instance, when we ask a friend ‘could you pass me the spoon’ and he does so, we assume that he is acting intentionally. However, in theory, it could be that our friend is possessed by a Cartesian evil demon guiding all his movements. This is a fanciful example just to highlight that, technically speaking, intentions can only be hypothesized. Hence, while it is not possible now to demonstrate intentionality in mice, it actually never will be. Nevertheless, what future research can do is search for an increasing number of pieces of evidence, a process that we hope this work will stimulate.

Additional research will be needed to corroborate our findings and further characterize this deceptive behaviour in mice. We hope that our work will spark interest for experimental research on intentional tactical deception in mice and open a conceptual debate on the topic. This would help to shed light not only on deception in non-human animals but also on non-human intentionality, a field of inquiry whose importance has often been highlighted by cognitive ethology [18,78–87]. Although it has been promoted and advocated by cognitive ethologists since the 1970s, the study of non-human animal intentionality is still underdeveloped, especially for what concerns non-primate species. Indeed, Dauphiné-Morer *et al.*, analyzing scientific articles published between 2016 and 2020, have recently reported how scarcely the term ‘intention’ is employed in non-human animal studies, and have pointed out the limitations of the current dominant theoretical framework used to study non-human animal cognition, which does not allow us to investigate and describe the full spectrum of non-human cognitive processes [87]. In the 1998 Ferrier Lecture, a prestigious lectureship on neuroscience topics held every three years by the Royal Society of London and established in 1928 to honour the memory of the British neurophysiologist David Ferrier (1843–1928), the influential French neurobiologist Jean-Pierre Changeux cautiously proposed that mice may possess ‘rudiments of intentionality’ [88]. Nevertheless, Changeux concluded that ‘the mouse cannot be a good animal model to investigate intentional relations and social understanding, where highest level is reached exclusively in humans with the theory of mind’ [88]. Since then, little has changed regarding the views on mouse intentionality. Only recently, Zhu & Kuchibhotla, from Johns Hopkins University, after testing mice in a multiple-trial two-choice task, concluded that mice are capable of making intentional choices and strategies [89]. Studying animals in semi-natural or natural environments offers the unique opportunity to observe spontaneous complex social interactions, increasing the likelihood to find possible evidence of intentional behaviour. For example, recently, the analysis of the multimodal communication and audience directedness of the greeting behaviour of the African elephants in the Jafuta Reserve in Zimbabwe provided evidence of intentionality in pachyderm communication [90]. Indeed, proposing new experimental designs to study social interactions of non-human animals, such as the one of the current work, could be useful to stimulate research in the still poorly studied area of non-human intentionality.

Behavioural testing of rodents in natural conditions through automated testing systems has already been performed successfully in the past, both for cognitive and motor functions. In particular, spatial memory of mice and rats in naturalistic enclosures has been assessed through giant radial maze-inspired structures [91–93], while voluntary-wheel running has been evaluated in free-living mice and rats through freely accessible running wheels [94,95] and frequency, as well as circadian patterns, of burrow visit activity has been measured in free-living mice by placing automated recording systems at the entrance of natural burrows [47].

In future research, the employment of ethologically based behavioural tests performed in nature on free-living animals could be extremely useful to open new windows on animal cognition. In particular, our paradigm using free-access test chambers to study spontaneous deceptive behaviours in the context of food competition could not only provide insights into unknown aspects of rodent psychology, but could also represent a new interesting animal model to look into mindreading-related social behaviours, as well as a methodology to investigate psychiatric disorders through an innovative neuroethological translational approach [96]. More generally, behavioural testing of rodents evaluated through test chambers placed in their natural environment could serve as context-enriched supplements to traditional laboratory behavioural methods [97]. Analogously, wild rodents could serve as heterozigosity-enriched alternatives to laboratory inbred rodent models [98]. Moreover, testing rodents in their natural habitats also preserves their network of relationships with other species, which in this way can be studied together. Indeed, often the comparison of the similarities and differences in the behaviour of two or more species, an approach known as comparative psychology [99], can elucidate the underlying psychological processes better than studying each species singularly. For instance, in our study tactical deception in *Apodemus* mice was observed in total in 22 cases, of which 21 performed by black-striped mice and 1 by a yellow-necked mouse. Considering that tactical deception is often performed by subordinates towards dominants [12,69,100–102] and that yellow-necked mice are bigger, stronger, more aggressive and dominant over other *Apodemus* species as black-striped mice and wood mice [103–107], it is not surprising to observe such disproportion in the frequency of deception. It appears that, since black-striped mice cannot overcome yellow-necked mice with strength, they instead employ tactics and deception to outperform their opponents avoiding a physical confrontation.

In the future, novel behavioural tests for free-living rodents, based on the free exploration paradigm [20,98,108,109], could be designed to evaluate currently unstudied behaviours or even behaviours that, being spontaneous and requiring complex contexts and/or social interactions, cannot be manifested by rodents in common laboratory settings.

## Data Availability

All data are available in the main text or in the supplementary materials [111].
